# Electronic Nicotine Vapor Exposure Produces Differential Changes in Central Amygdala Neuronal Activity, Thermoregulation and Locomotor Behavior in Male Mice

**DOI:** 10.1523/ENEURO.0189-21.2021

**Published:** 2021-08-10

**Authors:** M. Zhu, M. Echeveste Sanchez, E. A. Douglass, J. V. Jahad, T. D. Hanback, T. N. Guhr Lee, C. R. Esther, M. Cole, A. J. Roberts, M. A. Herman

**Affiliations:** 1 Department of Pharmacology, University of North Carolina at Chapel Hill, Chapel Hill, NC 27599; 2 Bowles Center for Alcohol Studies, University of North Carolina at Chapel Hill, Chapel Hill, NC 27599; 3 Neuroscience Curriculum, University of North Carolina at Chapel Hill, Chapel Hill, NC 27599; 4 Division of Pediatric Pulmonology, Department of Pediatrics, University of North Carolina at Chapel Hill, Chapel Hill, NC 27599; 5 Marsico Lung Institute/Cystic Fibrosis Research Center, University of North Carolina at Chapel Hill, Chapel Hill, NC 27599; 6 Animal Models Core, The Scripps Research Institute, La Jolla, CA 92037

**Keywords:** central amygdala, locomotion, neuronal firing, nicotine inhalation, thermoregulation, vapor exposure

## Abstract

Nicotine is an addictive substance historically consumed through smoking and more recently through the use of electronic vapor devices. The increasing prevalence and popularity of vaping prompts the need for preclinical rodent models of nicotine vapor exposure and an improved understanding of the impact of vaping on specific brain regions, bodily functions, and behaviors. We used a rodent model of electronic nicotine vapor exposure to examine the cellular and behavioral consequences of acute and repeated vapor exposure. Adult male C57BL/6J mice were exposed to a single 3-h session (acute exposure) or five daily sessions (repeated exposure) of intermittent vapes of 120 mg/ml nicotine in propylene glycol:vegetable glycerol (PG/VG) or PG/VG control. Acute and repeated nicotine vapor exposure did not alter body weight, and both exposure paradigms produced pharmacologically significant serum nicotine and cotinine levels in the 120 mg/ml nicotine group compared with PG/VG controls. Acute exposure to electronic nicotine vapor increased central amygdala (CeA) activity in individual neuronal firing and in expression of the molecular activity marker, cFos. The changes in neuronal activity following acute exposure were not observed following repeated exposure. Acute and repeated nicotine vapor exposure decreased core body temperature, however acute exposure decreased locomotion while repeated exposure increased locomotion. Collectively, these studies provide validation of a mouse model of nicotine vapor exposure and important evidence for how exposure to electronic nicotine vapor produces differential effects on CeA neuronal activity and on specific body functions and behaviors like thermoregulation and locomotion.

## Significance Statement

Nicotine vaping is increasing, prompting the need for an improved understanding of the impact of electronic nicotine vapor exposure on specific brain regions and relevant physiological functions and behaviors. The present study used a mouse model of nicotine vapor exposure to examine the cellular and behavioral consequences of acute and repeated exposure to nicotine vapor. We found that acute, but not repeated, exposure to nicotine vapor increased activity in the central amygdala (CeA) and that acute and repeated exposure produced differential effects on body temperature and movement. These findings demonstrate that nicotine vaping alters brain function in the CeA and produces dysregulation of normal body functions like thermoregulation and locomotion.

## Introduction

Nicotine, as a component of tobacco smoke or other nicotine delivery devices, is a highly addictive drug. Nicotine addiction is characterized by repeated cycles of intake culminating in the need for regular consumption and withdrawal symptoms during periods of abstinence (Markou, 2008). These behaviors appear to be mediated by central adaptations at the cellular level that can lead to long-lasting changes in structure and function of neurons and neuronal networks following repeated drug exposure and withdrawal (Lüthi and Lüscher, 2014). Repeated exposure to drugs of abuse has been shown to produce diminished effects over time, which can contribute to the development of tolerance and promote increases or maintenance of drug-seeking behavior. Although previous work has identified important effects of nicotine on central and peripheral function (Picciotto and Kenny, 2013; Picciotto and Mineur, 2014), studies integrating the effects of nicotine exposure via vapor inhalation are lacking, as are studies comparing how the effects of nicotine vapor exposure change over time. As the route of administration has been shown to have differential effects on the metabolism and pharmacokinetics of nicotine (Benowitz et al., 2009), it is imperative to consider the impact of route of administration in preclinical studies and studies examining the effects of nicotine via a nicotine vapor model are warranted.

Historically, the most common method for nicotine delivery in humans was the smoking of tobacco products like cigarettes. However, the number of current adult smokers has been steadily declining (Wang et al., 2018) and the use of electronic vapor (or e-vape) systems is increasing in prevalence and popularity (Palazzolo, 2013; Chaffee et al., 2017), particularly among younger populations, with an estimated 27.5% of high school students and 10.5% of middle schools students reporting current use of e-cigarettes (Cullen et al., 2019). Vaping is commonly thought of as having less associated health risks compared with tobacco smoking and has been suggested as a replacement method for smoking cessation (Palazzolo, 2013). However, recent studies have shown that vaping can produce cytotoxic effects on airway tissue (Lerner et al., 2015; Ghosh et al., 2018; Herman and Tarran, 2020), and the effects of vaping on neuronal function and addictive behaviors remain unclear. Recent studies using nicotine vapor models similar to the one used here have demonstrated that nicotine vapor exposure alters temperature regulation and locomotor function in rats (Javadi-Paydar et al., 2019; Lallai et al., 2021), produces nicotine-induced conditioned place preference (Frie et al., 2020), as well as spontaneous and mecamylamine-precipitated somatic signs of withdrawal (Montanari et al., 2020). Additionally, self-administration of nicotine vapor has recently been demonstrated in rodents (Smith et al., 2020; Cooper et al., 2021; Lallai et al., 2021) and it has been shown that self-administration of nicotine vapor can be enhanced with the addition of e-liquid flavors such as green apple and menthol (Cooper et al., 2021). Preclinical models using electronic nicotine vapor are an important tool to investigate cellular and brain region-specific mechanisms involved in the stages of electronic nicotine vapor exposure and the development of nicotine dependence. Whereas other models of nicotine exposure offer significant advantages like of the ability to deliver more precisely-timed systemic or intravenous nicotine dosages or voluntary oral consumption, electronic nicotine vapor exposure offers better translational relevance with regards to its real-world nicotine consumption by inhalation. However, the field at large suffers from an incomplete understanding of the basic parameters of electronic nicotine vapor exposure (i.e., nicotine concentration, exposure frequency/length, etc.) and the relevant cellular and behavioral consequences of different exposure paradigms.

Mechanistically, nicotine asserts its effects through the binding of nicotinic acetylcholine receptors, which are expressed throughout the nervous system (Picciotto and Mineur, 2014). A number of brain regions have been identified as targets of nicotine-induced plasticity, including the mesolimbic reward pathway and the amygdala (Adinoff, 2004). The central amygdala (CeA) is a central component of the limbic system and confers emotional relevance to internal and external sensory input to coordinate appropriate behavioral responses. In this context, the CeA has been implicated in numerous adaptive behaviors (feeding, fear learning, stress; Ciocchi et al., 2010; Gilpin et al., 2015; Douglass et al., 2017) and maladaptive conditions (anxiety, depression, chronic stress, addiction; Koob et al., 1998; Kenny et al., 2009; Gilpin et al., 2015; Bolton et al., 2018). Nicotine has been shown to produce variable effects on CeA activity and plasticity dependent on dose, timing, and route of administration (Brunzell et al., 2003; Valjent et al., 2004). Additionally, specific ensembles of neurons in the CeA have also been shown to contribute to the incubation of nicotine craving as evidenced by increased nicotine seeking following chronic intravenous nicotine self-administration and withdrawal (Funk et al., 2016). The CeA has been implicated in the central effects of nicotine, however the effects of acute and repeated nicotine vapor exposure on CeA electrophysiological activity and synaptic transmission remains understudied.

One of the primary goals of nicotine research is to understand how nicotine exposure impairs or dysregulates cellular functions to produce long-lasting maladaptive changes to brain circuitry and neuroplasticity. The present study utilizes a preclinical model of electronic nicotine vapor (e-vape) exposure to study the cellular and physiological consequences of acute and repeated exposure on CeA neuronal activity, thermoregulation, and locomotion.

## Materials and Methods

### Animals

For all experiments, adult male C57BL/6J mice (total *N* = 104; The Jackson Laboratory) were used. All mice were group housed in a temperature-controlled and humidity-controlled 12/12 h light/dark (7 A.M. lights on, 7 P.M. lights off) facility with *ad libitum* access to food and water and access to environmental enrichment. All experimental procedures were approved by the Institutional Animal Care and Use Committee.

### Drugs

(-)-nicotine free base and propylene glycol (PG) were purchased from Sigma. Vegetable glycerol (VG) was purchased from Fisher. DNQX (10 mm), AP-5 (50 mm), and CGP55845A (1 mm) were purchased from Tocris Bioscience.

### Electronic nicotine vapor delivery system

Mice were placed in chambers for vaporized delivery of 120 mg/ml (-)-nicotine free base (Sigma N3876) in a 30/70 (v/v) PG (Sigma P4347)/VG (Fisher G33-500) solution or PG/VG control solution. Either 120 mg/ml Nic or PG/VG solution was filled into e-vape tanks (Baby Beast Brother, Smok) that were then screwed into the vapor generator (95 W, Model SVS200, La Jolla Alcohol Research, Inc) that triggers the heating of the vape solution into vapor. The vape generator was connected to the e-vape controller (Model SSV-1, La Jolla Alcohol Research, Inc) that controls duration and frequency of vape delivery. The air-tight chambers are connected to a vacuum system that constantly pulls room air through the chambers at ∼1 l/min and ensures that each triggered vape is pulled into the chamber. Each 3-s vape puff takes ∼1 min to clear the chamber. Vape exposure sessions start between 9 and 10 A.M. during the animals’ light cycle, but vapor exposure was administered in the dark with lights off in the room. Repeated exposure was performed on consecutive days over the same approximate time period. After vape exposure, mice were returned to their home cage and regular housing facility.

### Serum analysis of nicotine and metabolites

Trunk blood from mice were collected immediately following acute or last session of repeated vape exposure. In animals used for slice electrophysiology experiments, trunk blood was collected following rapid decapitation. In animals used for cFos experiments, trunk blood was collected from cardiac puncture following pentobarbital (150 mg/kg, i.p.) injection before perfusion. Separate cohorts of animals were used for measuring nicotine and cotinine levels following a single 3 s 120 mg/ml nicotine vape (*N* = 6) and time course following acute vape (*N* = 4/time point). Trunk blood samples were spun down in a centrifuge and the serum layer was then collected and stored at −20°C before it was analyzed for nicotine and cotinine using liquid chromatography-tandem mass spectrometry (LC-MS/MS) as previously described (Ghosh et al., 2019).

### Slice electrophysiology

Immediately following acute electronic vapor exposure (PG/VG *N* = 6, Nic *N* = 6) or the last session of repeated electronic vapor exposure (PG/VG *N* = 6, Nic *N* = 6), mice were rapidly decapitated, and brains were extracted and placed into an ice-cold sucrose solution containing the following: 206.0 mm sucrose, 2.5 mm KCl, 0.5 mm CaCl_2_, 7.0 mm MgCl_2_, 1.2 mm NaH_2_PO_4_, 26 mm NaHCO_3_, 5.0 mm glucose, and 5 mm HEPES. Coronal slices (300 μm thick) containing the CeA were prepared with a Leica VT1000S (Leica Microsystems) and incubated in oxygenated (95% O_2_/5% CO_2_) artificial CSF (aCSF) containing the following: 120 mm NaCl, 2.5 mm KCL, 5 mm EGTA, 2.0 mm CaCl_2_, 1.0 mm MgCl_2_, 1.2 mm NaH_2_PO_4_, 26 mm NaHCO_3_, 1.75 mm glucose, and 5 mm HEPES for 30 min at 37°C, followed by 30-min equilibration at room temperature (RT; 20–22°C). For all recordings, patch pipettes (4–7 MΩ; King Precision Glass Inc.) were filled with internal solution containing the following: 145 mm KCl, 5 mm EGTA, 5 mm MgCl_2_, 10 mm HEPES, 2 mm Na-ATP, and 0.2 mm Na-GTP, and slices were superfused with oxygenated aCSF (described above). Cell firing was measured using the juxtacellular (cell-attached) configuration in gap-free voltage-clamp recording mode while membrane properties and spontaneous IPSCs (sIPSCs) were measured using whole-cell voltage-clamp (V_hold_ = −60 mV) recording modes in the presence of glutamate and GABA_B_ receptor antagonists (20 μm DNQX, 50 μm AP-5, 1 μm CGP55845A) to isolate GABA_A_ receptor currents. All recording data were acquired with Multiclamp 700B amplifier (Molecular Devices), low pass filtered at 2–5 kHz, digitized (Digidata 1440A; Molecular Devices), and stored on a computer using pClamp 10 software (Molecular Devices).

### Immunohistochemistry

Immediately following acute electronic vape exposure (PG/VG *N* = 8, Nic *N* = 8) or the last session of repeated electronic vapor exposure (PG/VG *N* = 9, Nic *N* = 9), mice were anesthetized with pentobarbital (150 mg/kg, i.p.) and perfused with 1× phosphate-buffered saline (PBS) followed by 4% paraformaldehyde (PFA) in PBS. Brains were postfixed in 4% PFA at 4°C overnight then transferred to 30% sucrose in PBS at 4°C until brains sank. Brains were serially sectioned at 40 μm using either a cryostat (Leica CM3050S, Leica Biosystems) or a microtome (HM450, Thermo Fisher Scientific) and slices were stored in 0.01% sodium azide in PBS at 4°C.

Four to five sections containing the CeA from each animal were selected for cFos immunoreactivity. Sections were designated as anterior (bregma −0.70 to −0.94), middle (bregma −1.06 to −1.34), or posterior (bregma −1.46 to −1.58) CeA using a mouse brain atlas (Franklin and Paxinos, 2008) as reference. All sections were washed in PBS for 10 min, then incubated with 50% methanol in PBS for 30 min, 3% hydrogen peroxide in PBS for 5 min, and blocking solution (0.3% Triton X-100; Thermo Fisher), 1% bovine serum albumin (BSA; Sigma) for 1 h, all at RT. Slices were then incubated at 4°C with rabbit anti-cFos primary antibody (1:3000, Millipore Sigma; ABE457) in blocking solution for 24–48 h. Sections were washed with Tris, NaCl, Triton X-100 (TNT) buffer and Tris, NaCl, blocking reagent (TNB; PerkinElmer) buffer then incubated in goat anti-rabbit horseradish peroxidase (HRP; 1:200, Abcam ab6721) in TNB buffer for 30 min followed by another round of TNT buffer washes. Fluorescence signal in the CeA were amplified by incubating in tyramide conjugated fluorescein (1:50) in TSA amplification diluent (Akoya Biosciences, NEL741001KT) for 10 min at RT. Slices were washed again with TNT buffer before being mounted onto slides using Vectashield (Vector labs; H1500) and coverslipped. Fluorescent signal in the CeA was detected and imaged on a fluorescent microscope (Nikon Eclipse 6600) under 20× objective.

### Body temperature

Core body temperatures were measured in mice (PG/VG *N* = 10, Nic *N* = 10) immediately on removal from the vapor chambers using a digital thermometer (Body Temperature Thermometer, 50316, Stoelting Co), with a mouse rectal probe (#RET; 3/4” length, 0.028” diameter; Braintree Scientific). Repeated measures were taken from the same animals following one 3-h vape session (acute), five 3-h vape sessions (repeated), and 72 h following repeated vape session (withdrawal).

### Open field locomotion

This test predicts how animals respond when introduced into a novel open arena and is used to capture spontaneous activity measures. The apparati are square white Plexiglas (50 × 50 cm) open fields illuminated to 360 lux in the center. Following body temperature assessment, each animal was placed in the center of the field and distance traveled and velocity were recorded during a 10-min observation period and analyzed using Noldus Ethovision XT software.

### Statistical analysis

Membrane characteristics and excitability cell-attached firing data were analyzed with Clampfit 10.6 (Molecular Devices). Frequency, amplitude, and decay of sIPSCs were analyzed and visually confirmed using a semiautomated threshold-based detection software (Mini Analysis). Electrophysiological data are reported as individual cell and averaged by animal and Grubb’s outlier test was used to find and remove outliers in datasets. Quantification of immunohistochemistry was performed in a blinded manner on two to six sections spanning anterior-posterior (AP) axis of CeA per animal using ImageJ. Statistical analysis of all experimental parameters was conducted using Prism 9.0 (GraphPad). Experimental parameters were analyzed and compared between groups using unpaired two-tailed *t* test, one-way, or two-way ANOVA, with Sidak’s repeated measures where appropriate. All data are expressed as mean ± SEM with *p* < 0.05 set as the threshold for statistical significance.

## Results

### Electronic nicotine vapor exposure paradigm

**Figure 1.**
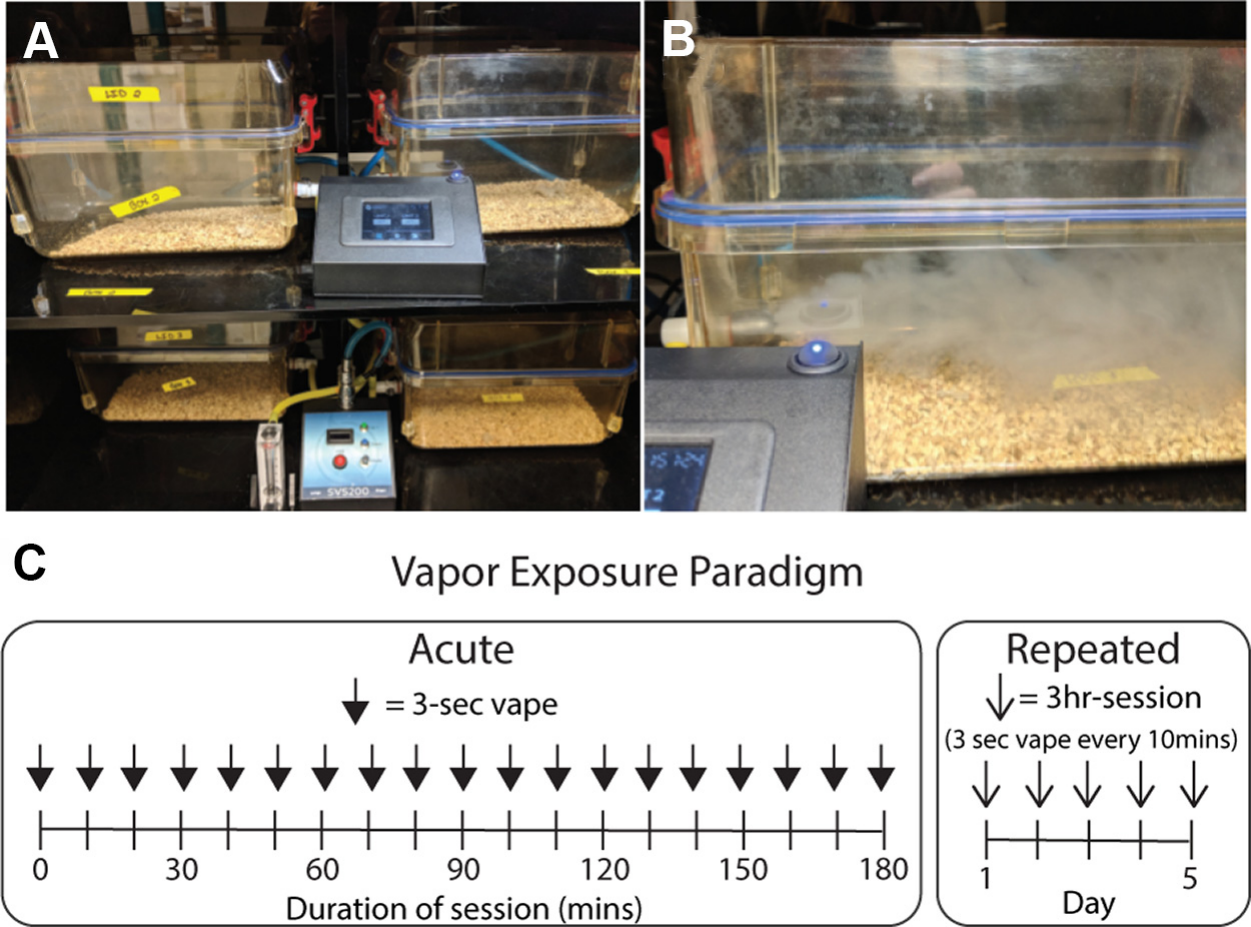
Mouse electronic nicotine vapor system, exposure parameters, and exposure paradigm (acute and repeated). ***A***, Mouse electronic nicotine vapor delivery system including vapor chambers, vapor generator, and e-vape controller. ***B***, Example of a 3-s vapor delivery inside the chamber. ***C***, Vapor exposure paradigm of acute (left) and repeated (right) exposure.

To establish a model of passive electronic nicotine vapor exposure in mice, we employed a commercially available system from La Jolla Alcohol Research, Inc comprised of vacuum-controlled chambers (Fig. 1*A*) where time-triggered vapes are delivered (Fig. 1*B*), and cleared from the chamber in approximately 1 min. To mimic the intermittent pattern of vaping in humans, we set the exposure parameters to deliver 3-s vapes with 10-min intervals between vapes. To investigate the difference following a single vape exposure versus multiple vape exposures, we placed mice (one to five per chamber) into the vape chambers (Fig. 1*A*,*B*) and exposed them to one of two vape paradigms, acute or repeated, respectively. In the acute vape exposure, mice were exposed to a 3-s vape every 10 min over a 3-h session (Fig. 1*C*, left). In the repeated vape exposure, mice were exposed to the same 3-h session, but for five consecutive days (Fig. 1*C*, right). In both acute and repeated exposure paradigms, mice were separated into two groups where one group was exposed to vehicle control PG/VG, and the other was exposed to 120 mg/ml nicotine in PG/VG.

### Body weight and nicotine metabolism following electronic nicotine vapor exposure

**Figure 2.**
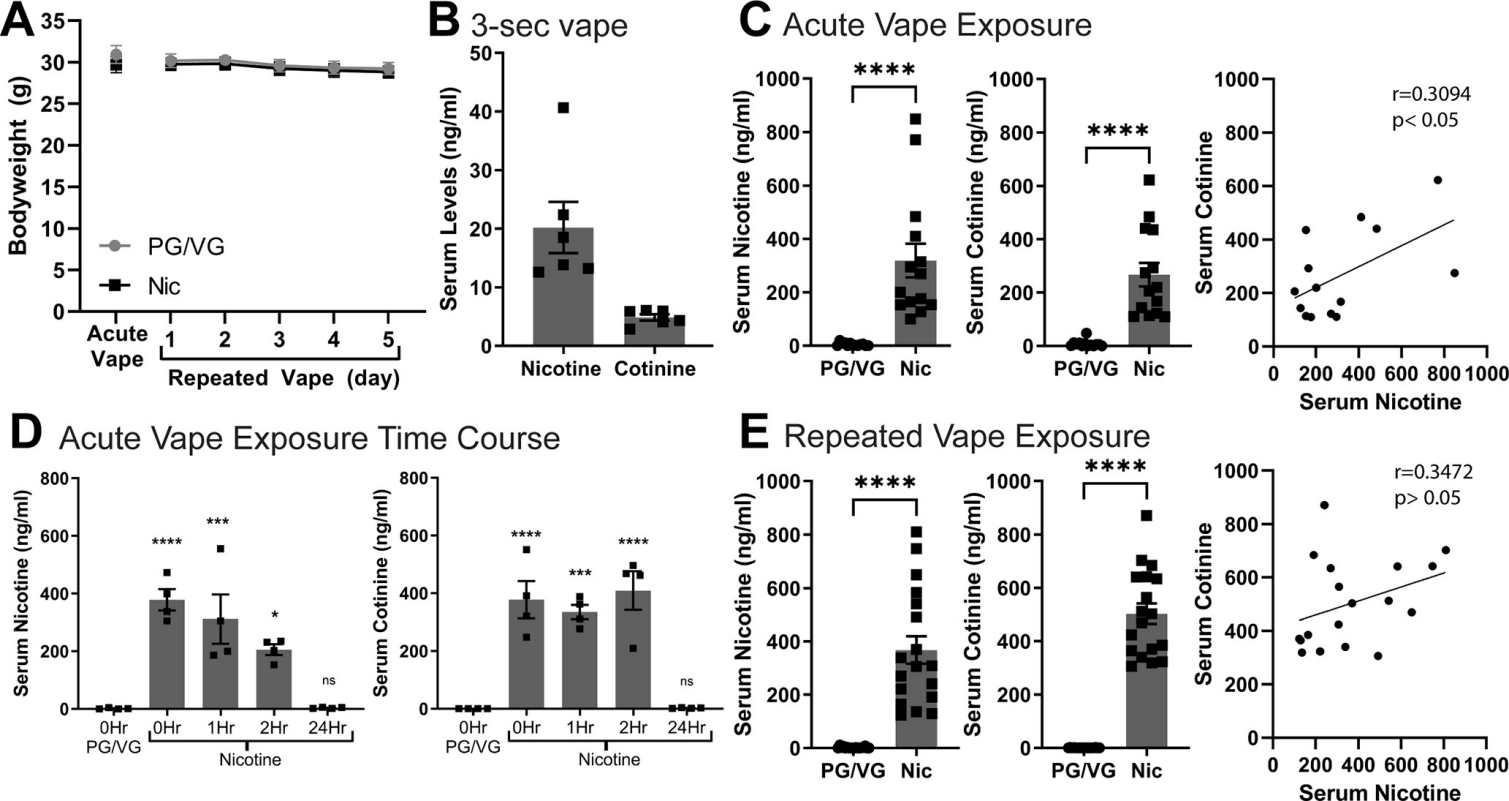
Serum nicotine and cotinine levels and bodyweight following acute and repeated electronic nicotine vapor exposure. ***A***, Body weights of animals exposed to acute (PG/VG *N* = 13, Nic *N* = 13) and repeated (PG/VG *N* = 12, Nic *N* = 12) vape (2-way ANOVA: main effect of day ****p < 0.0001). ***B***, Serum nicotine and cotinine levels following a single 3-s 120 mg/ml nicotine vape (*N* = 6). ***C***, Serum nicotine (left, unpaired *t*-test ****p < 0.0001) and cotinine (middle, unpaired *t*-test ****p < 0.0001) following acute PG/VG control (*N* = 13) or 120 mg/ml nicotine (*N* = 14) 3-h vape and correlation of serum nicotine and cotinine (right) ***D***, Time course of serum nicotine (left, 1-way ANOVA ****p < 0.0001; Dunnett’s compared to 0 Hr PG/VG: 0 Hr Nic ****p < 0.0001, 1 Hr Nic ***p < 0.0005, 2 Hr Nic *p < 0.05, 24 Hr Nic not significant) and cotinine (right, 1-way ANOVA ****p < 0.0001; Dunnett’s compared to 0 Hr PG/VG: 0 Hr Nic ****p < 0.0001, 1 Hr Nic ***p < 0.0005, 2 Hr Nic ****p < 0.0001, 24 Hr Nic not significant) following acute PG/VG or 120 mg/ml nicotine vape (PG/VG 0 h *N* = 4, Nic 0 h *N* = 4, Nic 1 h *N* = 4, Nic 2 h *N* = 4, Nic 24 h *N* = 4). ***E***, Serum nicotine (left, unpaired *t*-test ****p < 0.0001) and cotinine (middle, unpaired *t*-test ****p < 0.0001) following repeated PG/VG control (*N* = 18) or 120 mg/ml nicotine (*N* = 18) vape and correlation of serum nicotine and cotinine (right).

To examine the impact of acute and repeated nicotine vapor exposure on body weight, mice exposed to PG/VG control or 120 mg/ml nicotine were weighed daily over the course of the exposure paradigm. Following acute exposure, average body weights of PG/VG and nicotine groups were not significantly different (PG/VG 30.92 ± 1.08 g, *N* = 13; Nic 29.69 ± 0.94 g, *N* = 13; *t* = 0.8625, df = 24, *p* = 0.40, unpaired *t* test; Fig. 2*A*). In mice with repeated exposure, two-way ANOVA of body weight data showed no interaction of day × vape content (*F*_(4,88)_ = 0.04,896, *p* = 0.9954), no main effect of vape content (*F*_(1,22)_ = 0.1302, *p* = 0.7217), but a main effect of day (*F*_(2.653,58.36)_ = 19.47, ****p < 0.0001; Fig. 2*A*). However, a *post hoc* Sidak’s multiple comparison’s test show no significant differences between PG/VG and Nic groups for each day. This suggests that the body weight of mice exposed to 120 mg/ml nicotine was not significantly different from mice exposed to PG/VG control in both acute and repeated exposure paradigms and that electronic nicotine vapor exposure does not negatively impact the maintenance of body weight compared with PG/VG controls.

To examine nicotine metabolism following vapor exposure, we measured levels of serum nicotine and the nicotine metabolite cotinine. In a separate cohort of animals (*N* = 6), we found that following a single 3-s vape of 120 mg/ml nicotine, average serum nicotine was 20.21 ± 4.37 ng/ml and average serum cotinine was 4.862 ± 0.53 ng/ml (Fig. 2*B*). In mice exposed to an acute 3-h session of vape (PG/VG *N* = 13, Nic *N* = 14), serum nicotine and cotinine were significantly greater in the 120 mg/ml Nic group as compared with PG/VG controls [serum nicotine: PG/VG 4.938 ± 1.679 ng/ml, Nic 319.2 ± 63/93 ng/ml, *t* = 4.804, df = 25, ****p < 0.0001, unpaired *t* test (Fig. 2*C*, left); serum cotinine: PG/VG 8.045 ± 3.55 ng/ml, Nic 267.2 ± 44.29 ng/ml, *t* = 5.616, df = 25, ****p < 0.0001, unpaired *t* test (Fig. 2*C*, middle)]. Serum nicotine and serum cotinine levels from acute 120 mg/ml nicotine vaped animals were positively correlated (slope = 0.3916, intercept = 142.3, *r*_(12)_ = 0.5562, *p = 0.0389; Fig. 2*C*, right). In a separate cohort of mice, we measured serum nicotine and cotinine at 0, 1, 2, and 24 h, following an acute 3-h vape session (PG/VG 0 h *N* = 4, Nic 0 h *N* = 4, Nic 1 h *N* = 4, Nic 2 h *N* = 4, Nic 24 h *N* = 4). In comparison to the 0 h PG/VG control group, serum nicotine and cotinine were significantly greater in animals exposed to acute 120 mg/ml Nic at 0, 1, and 2 h, but this difference was eliminated at 24 h [serum nicotine: 0 h PG/VG 1.280 ± 0.97 ng/ml, 0 h Nic 378.6 ± 36.94 ng/ml, 1 h Nic 311.9 ± 85.44 ng/ml, 2 h Nic 205.6 ± 18.88 ng/ml, 24 h Nic 3.488 ± 1.102 ng/ml, *F*_(4,15)_ = 16.70, ****p < 0.0001, one-way ANOVA (Fig. 2*D*, left); serum cotinine 0 h PG/VG 0.2625 ± 0.08 ng/ml, 0 h Nic 377.9 ± 64.58 ng/ml, 1 h Nic 334.7 ± 25.08 ng/ml, 2 h Nic 409.2 ± 67.00 ng/ml, 24 h Nic 2.283 ± 0.57 ng/ml, *F*_(4,15)_ = 22.80, ****p < 0.0001, one-way ANOVA (Fig. 2*D*, right)]. Similar to acute exposure, mice exposed to repeated vape exposure (PG/VG *N* = 18, Nic *N* = 18) showed significantly greater serum nicotine and cotinine in the 120 mg/ml Nic group as compared with PG/VG controls [serum nicotine: PG/VG 2.06 ± 0.74 ng/ml, Nic 346.0 ± 51.40 ng/ml, *t* = 7.119, df = 34, ****p < 0.0001, unpaired *t* test (Fig. 2*E*, left); serum cotinine: PG/VG 0.59 ± 0.15 ng/ml, Nic 502.9 ± 38.63 ng/ml, *t* = 13.00, df = 34, ****p < 0.0001 (Fig. 2*E*, middle)]. Serum nicotine and serum cotinine levels from repeated 120 mg/ml nicotine vaped animals had a positive relationship but were not significantly correlated (slope = 0.2609, intercept = 406.9, *r*_(16)_ = 0.3472, *p* = 0.1581; Fig. 2*E*, right). Taken together, these data show that animals exposed to acute and repeated 120 mg/ml nicotine have higher serum nicotine and cotinine levels as compared with its PG/VG control groups.

### Effects of acute electronic nicotine vapor exposure on CeA neuron activity

**Figure 3.**
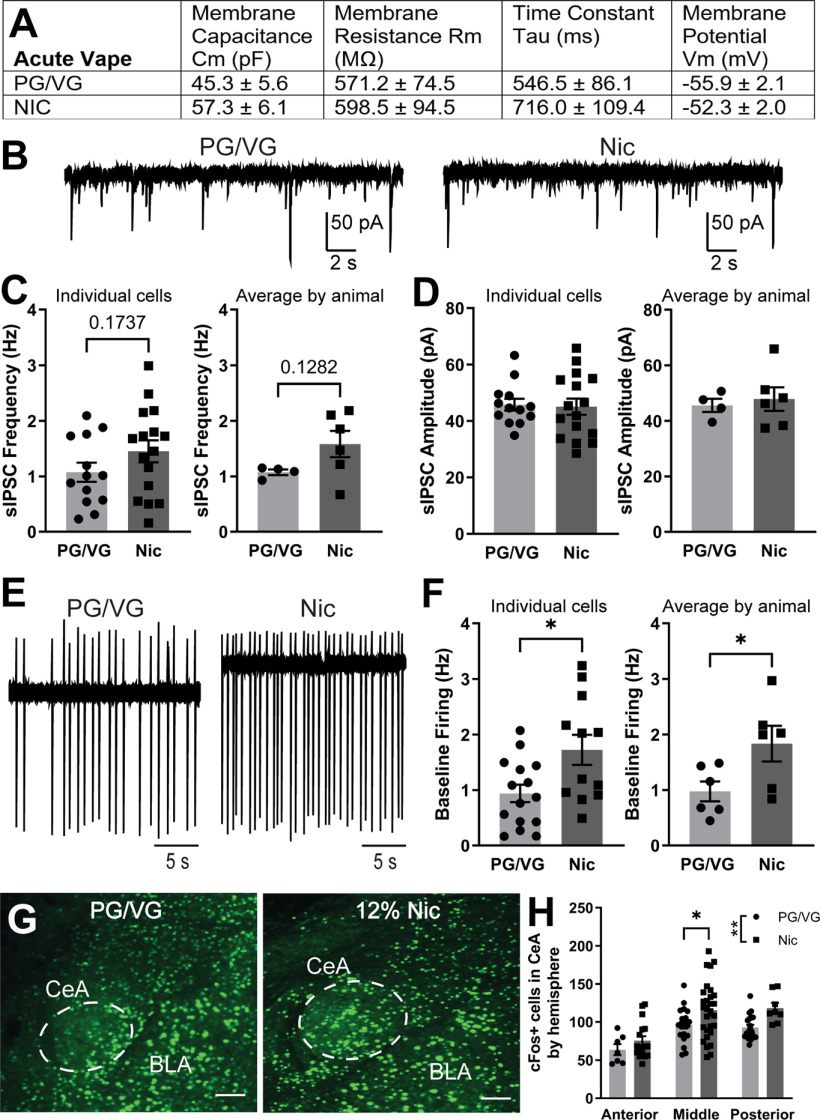
Inhibitory transmission and neuronal activity in the CeA following acute electronic nicotine vapor exposure. ***A***, Membrane properties of CeA neurons from mice exposed to acute PG/VG control (*N* = 6 animals, *n* = 15 cells) or 120 mg/ml nicotine (*N* = 5 animals, *n* = 17 cells) vape. ***B***, Representative traces of sIPSCs in CeA neurons from mice exposed to acute PG/VG control (left) or 120 mg/ml nicotine vape (right). ***C***, Summary of sIPSC frequency by individual cells (left) and averaged by animal (right) in CeA neurons from mice exposed to acute PG/VG (*N* = 4 animals, *n* = 13 cells) or 120 mg/ml nicotine (*N* = 6 animals, *n* = 16 cells) vape. ***D***, Summary of sIPSC amplitude by individual cells (left) and averaged by animal (right) in CeA neurons from mice exposed to acute PG/VG (*N* = 4 animals, *n* = 13 cells) or 120 mg/ml nicotine (*N* = 6 animals, *n* = 16 cells) vape. ***E***, Representative traces of cell-attached firing in CeA neurons from mice exposed to acute PG/VG control (left) or 120 mg/ml nicotine vape (right). ***F***, Summary of cell-attached firing frequency by individual cells (left, unpaired *t*-test *p < 0.05) and averaged by animal (right, unpaired *t*-test *p < 0.05) in CeA neurons from mice exposed to acute PG/VG (*N* = 6 animals, *n* = 15 cells) or 120 mg/ml nicotine (*N* = 6 animals, *n* = 12 cells) vape. ***G***, Representative micrograph of fluorescently labeled cFos in the CeA of mice exposed to acute PG/VG or 120 mg/ml nicotine vape. Scale bar: 100 μm. ***H***, Summary of cFos-positive cells by hemisphere across anterior to posterior CeA of mice exposed to acute PG/VG (*n* = 48 hemispheres in 8 animals) or 120 mg/ml nicotine (*n* = 54 hemispheres in 8 animals) vape (2-way ANOVA: main effect of AP ****p < 0.0001 and vape content **p < 0.01; Sidak’s Middle CeA *p < 0.05).

Once we established an electronic nicotine vape exposure model, we next investigated the impact of acute vape exposure on neuronal activity in the CeA using both electrophysiological and immunohistochemical techniques. First, we examined the membrane properties of CeA neurons from PG/VG and 120 mg/ml Nic-exposed male mice. Membrane capacitance, membrane resistance, time constant, and membrane potential were not statistically different between the two vape groups by unpaired *t* test (Fig. 3*A*). We then examined inhibitory synaptic transmission by measuring sIPSCs in CeA neurons from PG/VG and 120 mg/ml Nic mice and found no significant difference in sIPSC frequency by individual cells (PG/VG 1.08 ± 0.17 Hz, *n* = 13 cells; Nic 1.45 ± 0.20 Hz, *n* = 16 cells; *t* = 1.397, df = 27, *p* = 0.1737, unpaired *t* test; Fig. 3*C*, left) or averaged by animal (PG/VG 1.08 ± 0.05 Hz, *N* = 4 animals; Nic 1.58 ± 0.24 Hz, *N* = 6 animals; *t* = 1.697, df = 8, *p* = 0.1282, unpaired *t* test; Fig. 3*C*, right). Similarly, we found no significant difference in sIPSC amplitude by individual cells (PG/VG 45.81 ± 2.10 pA, *n* = 13 cells, Nic 45.11 ± 2.88 pA, *n* = 16 cells; *t* = 0.1879, df = 27, *p* = 0.8524, unpaired *t* test; Fig. 3*D*, left) or averaged by animal (PG/VG 45.65 ± 2.37 pA, *N* = 4 animals; Nic 47.91 ± 4.24 pA, *N* = 6 animals; *t* = 0.4024, df = 8, *p* = 0.698, unpaired *t* test; Fig. 3*D*, right). However, when we examine cell firing, we found a significantly greater baseline firing rate in CeA neurons from the 120 mg/ml Nic group compared with PG/VG controls when analyzed by individual cell (PG/VG 0.94 ± 0.16 Hz, *n* = 15 cells; Nic 1.73 ± 0.23 Hz, *n* = 12 cells; *t* = 2.638, df = 25, *p = 0.0141, unpaired *t* test; Fig. 3*F*, left) and average by animal (PG/VG 0.98 ± 0.18 Hz, *N* = 6 animals; Nic 1.84 ± 0.32 Hz, *N* = 6 animals; *t* = 2.340, df = 10, *p = 0.0414, unpaired *t* test; Fig. 3*F*, right). We then examined neuronal activity across the AP span of the CeA by immunohistochemical labeling of cFos, an immediate early gene marker for cell activity. Two-way ANOVA revealed a main effect of AP coordinates (*F*_(2,96)_ = 14.57, ****p < 0.0001) and vape content (*F*_(1,96)_ = 9.942, **p = 0.0022; Fig. 3*H*). *Post hoc* Sidak’s multiple comparison test found that the middle CeA of mice exposed to 120 mg/ml Nic possessed a greater number of cFos-positive cells than that of mice exposed to PG/VG control vape (*p = 0.0384; Fig. 3*H*). This is consistent with our CeA firing data following acute vape (Fig. 3*F*) as a majority of the electrophysiological recordings are from cells in the middle CeA. Taken together, these data indicate that exposure to an acute 3-h session of 120 mg/ml Nic electronic vapor increases activity of CeA neurons as compared with PG/VG controls.

### Effects of repeated electronic nicotine vapor exposure on CeA neuron activity

**Figure 4.**
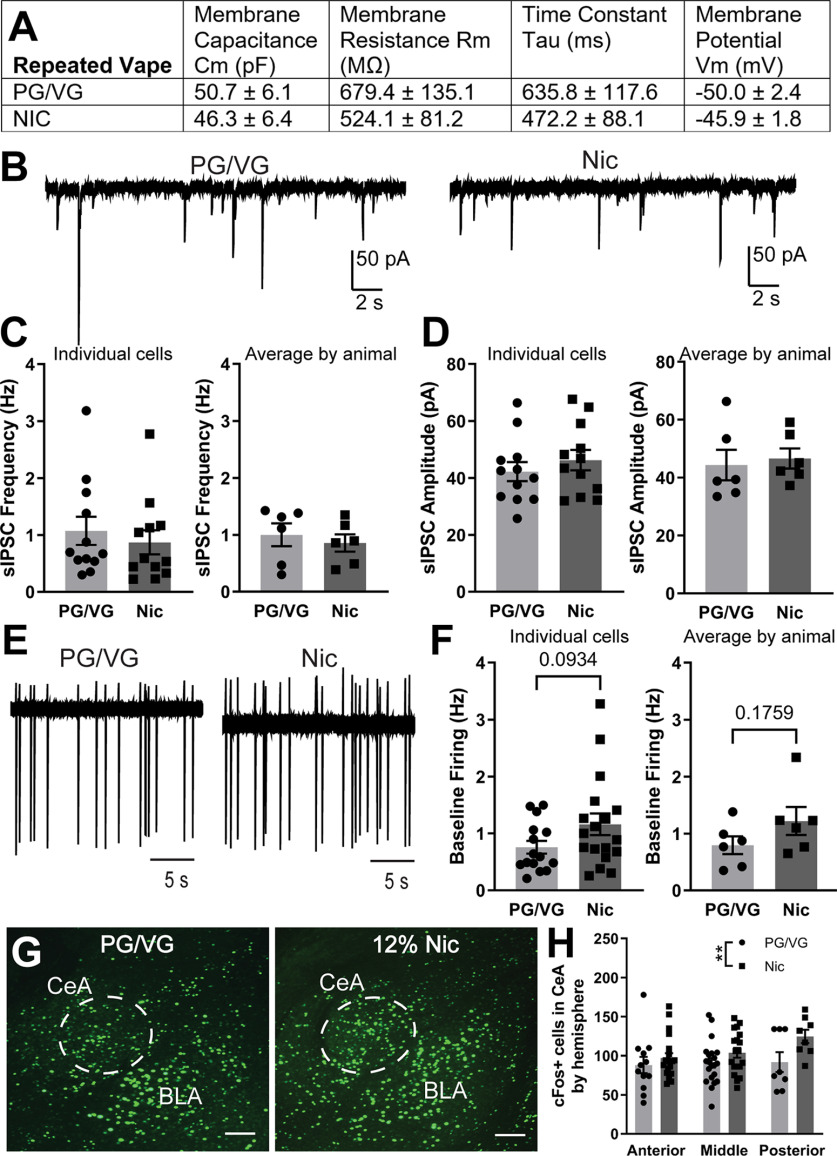
Inhibitory transmission and neuronal activity in the CeA following repeated electronic nicotine vapor exposure. ***A***, Membrane properties of CeA neurons from mice exposed to repeated PG/VG control (*N* = 6 animals, *n* = 17 cells) or 120 mg/ml nicotine (*N* = 6 animals, *n* = 18 cells) vape. ***B***, Representative traces of sIPSCs in CeA neurons from mice exposed to repeated PG/VG control (left) or 120 mg/ml nicotine vape (right). ***C***, Summary of sIPSC frequency by individual cells (left) and averaged by animal (right) in CeA neurons from mice exposed to repeated PG/VG (*N* = 6 animals, *n* = 12 cells) or 120 mg/ml nicotine (*N* = 5 animals, *n* = 12 cells) vape. ***D***, Summary of sIPSC amplitude by individual cells (left) and averaged by animal (right) in CeA neurons from mice exposed to repeated PG/VG (*N* = 6 animals, *n* = 12 cells) or 120 mg/ml nicotine (*N* = 5 animals, *n* = 12 cells) vape. ***E***, Representative traces of cell-attached firing in CeA neurons from mice exposed to repeated PG/VG control (left) or 120 mg/ml nicotine vape (right). ***F***, Summary of cell-attached firing frequency by individual cells (left) and averaged by animal (right) in CeA neurons from mice exposed to repeated PG/VG (*N* = 6 animals, *n* = 15 cells) or 120 mg/ml nicotine (*N* = 6 animals, *n* = 18 cells) vape. ***G***, Representative micrograph of fluorescently labeled cFos in the CeA of mice exposed to repeated PG/VG or 120 mg/ml nicotine vape. Scale bar: 100 μm. ***H***, Summary of cFos-positive cells by hemisphere across anterior to posterior CeA of mice exposed to repeated PG/VG (*n* = 41 hemispheres in 9 animals) or 120 mg/ml nicotine (*n* = 47 hemispheres in 9 animals) vape (2-way ANOVA: main effect of vape content **p < 0.01).

Previous research has shown that repeated exposure to nicotine can cause differential changes in synaptic transmission and/or neuronal activity in a number of brain regions (De Biasi and Dani, 2011; Picciotto and Mineur, 2014). Here, we examined how CeA activity is changed following repeated (5 d) exposure to 120 mg/ml nicotine electronic vapor. We first examined the membrane properties of CeA neurons from PG/VG and 120 mg/ml Nic-exposed mice and found that membrane capacitance, membrane resistance, time constant, and membrane potential were all not significantly different between the two groups (Fig. 4*A*). We then examined inhibitory transmission in CeA neurons and found no significant difference in sIPSC frequency by individual cells (PG/VG 1.07 ± 0.25 Hz, *n* = 12 cells; Nic 0.87 ± 0.21 Hz, *n* = 12 cells; *t* = 0.6145, df = 22, *p* = 0.5452, unpaired *t* test; Fig. 4*C*, left) or averaged by animal (PG/VG 1.00 ± 0.20 Hz, *N* = 6 animals; Nic 0.86 ± 0.15 Hz, *N* = 6 animals; *t* = 0.5681, df = 10, *p* = 0.5825, unpaired *t* test; Fig. 4*C*, right). Similarly, we found no significant difference in sIPSC amplitude by individual cells (PG/VG 42.24 ± 3.35 pA, *n* = 12 cells, Nic 46.31 ± 3.58 pA, *n* = 12 cells; *t* = 0.8301, df = 22, *p* = 0.4154, unpaired *t* test; Fig. 4*D*, left) or averaged by animal (PG/VG 44.38 ± 5.26 pA, *N* = 6 animals; Nic 46.66 ± 3.47 pA, *N* = 6 animals; *t* = 0.3626, df = 10, *p* = 0.7244, unpaired *t* test; Fig. 4*D*, right). We also found no significant difference in baseline firing between the PG/VG and 120 mg/ml Nic groups when analyzed by individual cell (PG/VG 0.76 ± 0.11 Hz, *n* = 15 cells; Nic 1.16 ± 0.19 Hz, *n* = 18 cells; *t* = 1.731, df = 31, *p* = 0.0934, unpaired *t* test; Fig. 4*F*, left) and average by animal (PG/VG 0.80 ± 0.16 Hz, *N* = 6 animals; Nic 1.22 ± 0.25 Hz, *N* = 6 animals; *t* = 1.457, df = 10, *p* = 0.1759, unpaired *t* test; Fig. 4*F*, right). When we examined cFos expression across AP CeA, two-way ANOVA analysis revealed a main effect of vape content (*F*_(1,82)_ = 7.219, **p = 0.0087; Fig. 4*H*). However, *post hoc* Sidak’s test revealed no significant differences between PG/VG and 120 mg/ml Nic groups in anterior, middle, or posterior CeA (Fig. 4*H*), which is consistent with the electrophysiological data. Together, these data suggest that in contrast to the effects observed with acute exposure, repeated exposure to 120 mg/ml Nic vapor does not result in increased CeA activity as compared with PG/VG controls.

### Body temperature and locomotion following acute and repeated electronic nicotine vapor exposure

**Figure 5.**
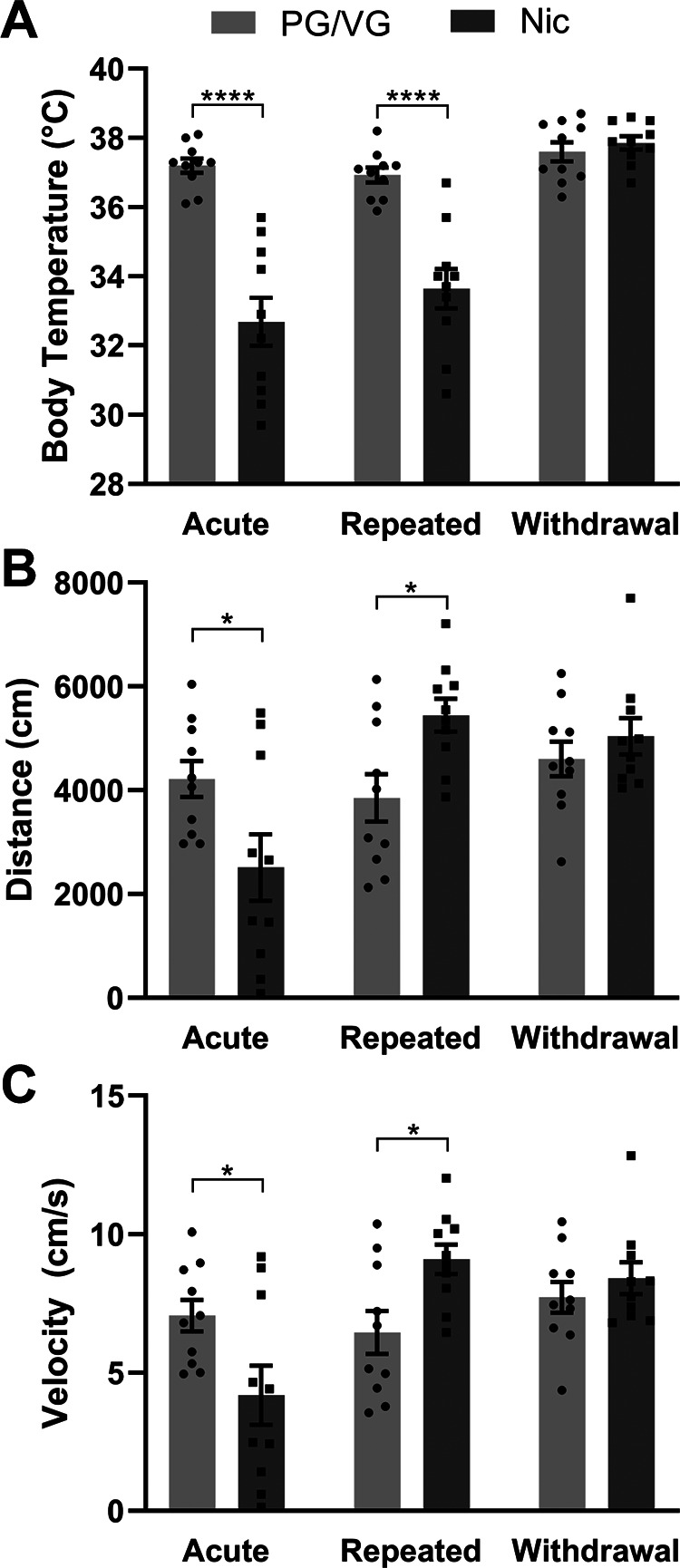
Body temperature and locomotion following acute and repeated electronic nicotine vapor exposure. ***A***, Body temperature of mice following acute, repeated, or withdrawal (2-way ANOVA interaction ****p < 0.0001: main effect of exposure schedule ****p < 0.0001 and vape content ****p < 0.0001; Sidak’s acute ****p < 0.0001, repeated ****p < 0.0001) from PG/VG or 120 mg/ml nicotine vapor exposure (PG/VG *N* = 10, Nic *N* = 10, repeated measures). ***B***, Distance traveled in open field assay in mice following acute, repeated, or withdrawal (2-way ANOVA interaction ****p < 0.0001: main effect of exposure schedule ****p < 0.0001; Sidak’s acute *p < 0.05, repeated *p < 0.05) from PG/VG or 120 mg/ml nicotine vapor exposure (PG/VG *N* = 10, Nic *N* = 10, repeated measures). ***C***, Locomotor velocity in open field assay in mice following acute, repeated, or withdrawal (2-way ANOVA interaction ****p < 0.0001: main effect of exposure schedule ****p < 0.0001; Sidak’s acute *p < 0.05, repeated *p < 0.05) from PG/VG or 120 mg/ml nicotine vapor exposure (PG/VG *N* = 10, Nic *N* = 10, repeated measures).

After identifying the metabolic, molecular, and cellular changes following acute and repeated electronic nicotine vapor exposure, we examined the *in vivo* impact of electronic nicotine vapor after a single acute session, after repeated 5-d sessions, or 72 h after the final session of the repeated sessions (withdrawal). As nicotine has been reported to have hypothermic effects (Javadi-Paydar et al., 2019), we first measured body temperature of mice immediately following vape exposure. Two-way ANOVA revealed an interaction between exposure schedule × vape content (*F*_(2,36)_ = 16.58, ****p < 0.0001) as well as main effects of exposure schedule (*F*_(2,36)_ = 24.90, ****p < 0.0001) and vape content (*F*_(1,18)_ = 68.24, ****p < 0.0001) alone. *Post hoc* Sidak’s multiple comparisons test revealed significantly lower body temperature in acute Nic (****p < 0.0001) and repeated Nic (****p < 0.0001), but not in withdrawal, as compared with PG/VG controls (PG/VG *N* = 10 animals, Nic *N* = 10 animals; Fig. 5*A*). We then assessed locomotor activity using the open field test. In both parameters measured (distance and velocity), two-way ANOVA revealed an interaction between exposure schedule × vape content (distance *F*_(2,36)_ = 14.89, ****p < 0.0001; velocity *F*_(2,36)_ = 14.83, ****p < 0.0001) and a main effect of exposure schedule (distance *F*_(2,36)_ = 13.48, ****p < 0.0001; velocity *F*_(2,36)_ = 13.50, ****p < 0.0001). *Post hoc* Sidak’s multiple comparisons test revealed significantly less distance traveled (****p < 0.0001) and at a slower velocity (*p = 0.0175) in acute Nic as compared with PG/VG controls (Fig. 5*B*). However, the opposite was revealed in repeated Nic with significantly more distance traveled (****p < 0.0001) and at a faster velocity (*p = 0.0315) as compared with PG/VG controls (PG/VG *N* = 10 animals, Nic *N* = 10 animals; Fig. 5*C*). In both distance and velocity measures for mice in withdrawal (72 h after repeated exposure), 120 mg/ml Nic was not significantly different from PG/VG controls. Collectively, these data demonstrate that acute and repeated exposure to 120 mg/ml nicotine vapor produce consistent hypothermic effects but divergent effects on locomotion, both of which are reversed in withdrawal.

## Discussion

These studies used a preclinical model of electronic nicotine vapor exposure to examine the efficiency and reliability of modeling electronic vapor exposure in male mice and to examine the effects of acute (single session) and repeated (five daily sessions) nicotine vape exposure. The data presented here demonstrate that male mice will tolerate electronic nicotine vapor exposure sessions characterized by repeated intermittent vapes and that repeated intermittent vaping results in significant nicotine levels in the blood and produces exposure paradigm-specific neuronal and behavioral effects. Specifically, acute exposure to electronic nicotine vapor produced significant increases in CeA activity that were not observed following repeated exposure. Peripherally, significant decreases in core body temperature were observed in male mice exposed to both acute and repeated electronic nicotine vapor session. Decreased locomotion was observed following acute but increased locomotion was observed following repeated electronic nicotine vapor exposure. Collectively, these data provide evidence for the utility of vapor exposure models in mice and demonstrate how both central and peripheral systems are differentially affected by both acute and repeated electronic nicotine vapor exposure.

The electronic nicotine vapor exposure method used in these studies has several notable advantages over previous models, but also some limitations that should be considered. One advantage of the electronic nicotine vapor exposure paradigm is that it mimics the delivery method used by humans, in that the nicotine is delivered in vapor form and the route of delivery is through inhalation. This model also mimics the administration method seen in social situations in which electronic cigarettes are common. Route of administration of nicotine is an important determining factor in the timing and magnitude of the reinforcing effects as well as the behavioral effects of nicotine exposure. In previous studies nicotine has been administered through experimenter-delivered injections (Kasten et al., 2016) or subcutaneous minipumps (LeSage et al., 2002). These models provide the benefit of standardized serum nicotine levels; however, these have limitations related to surgery and/or injection induced stress. A voluntary route of nicotine delivery is oral consumption with bottle choice which showed nicotine-induced hypothermic and locomotor effects (Kasten et al., 2016; O’Rourke et al., 2016; DeBaker et al., 2020). However, this model of nicotine exposure does not replicate human consumption of nicotine and raises questions regarding taste preference when paired with other substances like sucrose. Nicotine administered via inhalation of vapor has become an emerging route of human consumption thus, preclinical studies should aim to reflect this route of administration.

The experimental parameters were chosen to mimic the experience of vaping which is characterized by brief periods of nicotine vapor inhalation (i.e., “puffs”) interspersed with periods of regular air inhalation. The 3-s vape period was chosen as it was sufficient to fill the cage with vapor and was consistent with vape duration of a puff in human e-cig users (Dawkins et al., 2016; Hiler et al., 2017). Studies in humans have also found that when given the *ad libitum* vape over the course of 1 h, experienced male vapers have an average number of 48 puffs (Dawkins et al., 2016). In our paradigm, we have chosen the longer interval of 10 min between vape deliveries to mimic early recreational use with limited time periods. This also allow for clearance of the previous vape and to provide a period of regular air inhalation between vapes. Finally, the 3-h group exposure was chosen to model nicotine use that typically occurs in discrete time periods of exposure.

One important issue to consider in electronic nicotine vapor exposure paradigms is dosage. Commercial e-liquids offer a variety of nicotine concentrations, ranging from 3, 6, 12, 18, or 36 mg/ml. Studies in humans have found that a puff inhaled by an experienced e-cig user, as compared with a native e-cig user, is longer in duration and larger in volume and thus produce higher plasma nicotine levels. This effect was observed across multiple nicotine concentrations (Hiler et al., 2017) and emphasized how nicotine consumption via inhalation can be impacted by the variability in vapor topography. The 120 mg/ml nicotine concentration was chosen based on a 10-fold increase in what is found in commercial e-liquids (12 mg/ml) to account for the reduction in vapor volume as it is passively inhaled from chamber air as opposed to direct inhalation into the airway as observed with humans. This concentration was further validated by the pharmacologically relevant serum nicotine and cotinine levels (Fig. 2) and consistent with cotinine levels observed in human heavy smokers (Lawson et al., 1998). Previous preclinical studies have used lower nicotine vapor concentrations (1–80 mg/ml) with lower serum nicotine and cotinine levels (Javadi-Paydar et al., 2019; Frie et al., 2020; Montanari et al., 2020; Smith et al., 2020; Cooper et al., 2021; Lallai et al., 2021); however, lower concentrations have produced variable effects. Although 120 mg/ml nicotine concentration was relatively high compared with previous work and human vape products, future studies will compare serum nicotine levels at different nicotine concentrations. Another consideration of the current approach is the use of passive exposure, which allows for precise control of experimental parameters, but does not account for the volitional aspect of nicotine intake. As so little is known about the impact of electronic vapor exposure in mice, passive exposure was preferable for the direct comparison of cellular and behavioral consequences of electronic nicotine vapor exposure without the confounding factor of variable intake or timing of exposure. However, since voluntary administration remains an important aspect of studying volition in drugs of abuse and self-administration of drugs delivered by vapor inhalation in rodents has been demonstrated with nicotine (Smith et al., 2020; Cooper et al., 2021; Lallai et al., 2021), cannabis (Freels et al., 2020; Glodosky et al., 2020), and heroin (Gutierre et al., 2020), future studies will examine cellular and behavioral consequences of voluntary nicotine vapor self-administration to explore the impact of volition.

One important caveat to the current study is that all experiments were performed only in male mice, precluding our ability to identify any relevant sex differences in electronic nicotine vapor exposure. As females represent a significant proportion of the current vaping population [National Center for Chronic Disease Prevention and Health Promotion (US) Office on Smoking and Health, 2016], studies examining the impact of nicotine vapor exposure in female subjects are important and warranted. Previous studies in rats have demonstrated sex differences in nicotine metabolism (Kyerematen et al., 1988) and the effects of nicotine on anxiety-like behaviors (Torres et al., 2013). A recent study examining nicotine vapor self-administration in male and female rats identified that while both males and female rats will self-administer nicotine vapor in roughly equivalent levels, passive vapor exposure produced significantly lower serum cotinine levels in females as compared with males (Lallai et al., 2021). In addition, male rats displayed an increase in locomotion following repeated passive nicotine vapor exposure, while females did not, suggesting the potential for sex differences in behavioral sensitivity to passive nicotine vapor. Collectively, these data suggest that there may be important sex differences in the effects of nicotine vapor exposure in mice, which will be the subject of future studies.

The CeA has previously been implicated in the central effects of nicotine, however results were variable and largely dependent on dose, model, and timing of exposure. Exposure to a single 3-h session of electronic nicotine vapor exposure resulted in significant increases in CeA neuronal activity as measured by electrophysiological assessment of neuronal firing and by immunohistochemical assessment of the activity marker cFos. The CeA is a primarily GABAergic nucleus composed of interneurons and projection neurons (Pitkänen and Amaral, 1994) and inhibitory microcircuits within the CeA have been implicated in fear learning (Haubensak et al., 2010) and in the plasticity observed with acute and chronic ethanol exposure (Herman et al., 2016). However, the inhibitory inputs onto CeA neurons (as measured by sIPSCs) do not appear to be modulated by acute or repeated electronic nicotine vapor exposure, and the increases in neuronal activity following acute exposure were observed independent of changes in inhibitory signaling. In contrast to what was observed with acute exposure, following 5 d of repeated electronic nicotine vapor sessions, there was no increase in CeA activity observed by either electrophysiological or immunohistochemical evaluation. We used both electrophysiological (firing) and immunohistochemical (cFos) measures of neuronal activity in the CeA to examine single cell- versus population-specific changes in CeA activity following electronic nicotine vapor exposure. Our results from the two measures show similar direction of change in acute exposure and no change in repeated exposure.

These neuronal activity findings are consistent with previous studies where an increase in CeA cFos was found following a single subcutaneous injection of acute nicotine, but no increase in CeA cFos was observed following chronic nicotine exposure using osmotic minipumps. However, they also found increased cFos with a single subcutaneous nicotine injection following chronic osmotic minipump nicotine exposure (Salminen et al., 1999). Increased CeA cFos was also observed after 14 d of withdrawal from chronic nicotine self-administration (Funk et al., 2016). A single intraperitoneal injection of nicotine increased phosphorylated extracellular regulated kinase (pERK) in the CeA 20 min after drug administration (Valjent et al., 2004), however a study of voluntary nicotine drinking found no increase in amygdala pERK after an acute (1.3 h) drinking session and only saw a significant increase in amygdala pERK after chronic (28–30 d) drinking (Brunzell et al., 2003). Taken together, these studies suggest that the CeA is differentially engaged with different nicotine exposure models and at different timepoints of exposure and withdrawal.

The electronic nicotine vapor exposure system used in this study has also been employed in prior work examining the impact of electronically-generated nicotine vapor in rodents. One study in rats reported decreased core body temperature following nicotine vapor exposure (Javadi-Paydar et al., 2019), which is consistent with our findings as well as what has been shown following nicotine intraperitoneal injection (Javadi-Paydar et al., 2019) and subcutaneous injection (Levin et al., 2003; Akinola et al., 2019) and suggest that nicotine’s effect on body temperature are similar across rodent species and route of nicotine administration. Additionally, the decrease in locomotion we observed with acute nicotine vapor is consistent with studies where nicotine was delivered through intraperitoneal or subcutaneous injections (Levin et al., 2003; Akinola et al., 2019). Interestingly, the same rat study that reported similar decreases in core body temperature found no difference in locomotion between PG/VG and nicotine groups following a single 30 min vapor exposure (Javadi-Paydar et al., 2019). This divergence from our findings may be because of the difference in timing and duration of vape exposure of experiments compared with ours (single 30-min vape vs 3-h session of 3-s vape every 10 min, respectively) or the location of locomotor activity test (inhalation chamber vs novel open arena, respectively). However, following repeated nicotine vapor, increased locomotion was observed in multiple studies (Javadi-Paydar et al., 2019; Lallai et al., 2021), which is consistent with our findings suggesting another parameter that is consistent across rodent species.

Collectively, these studies provide important evidence for how acute and repeated exposure to electronic nicotine vapor can produce differential effects in the CeA and on specific behaviors. The development and more wide-spread use of preclinical models of electronic nicotine vapor exposure will allow for more detailed studies on the impact of vaping on additional brain regions and behaviors that could lead to an improved understanding of how vaping effects the human brain to promote the development of nicotine dependence specific to the vaping route of delivery. The importance of this work is underscored by the increasing prevalence of nicotine vaping and the prevailing assumption that since vaping represents a safer alternative to tobacco smoking, it is less “dangerous” or harmful of an activity. It will be important for scientific research to continue apace with human user experience so that neurobiological underpinnings of clinically-relevant nicotine vapor exposure models can be used to understand the impacts of vaping on human populations.

## Synthesis

Reviewing Editor: Alexxai Kravitz, Washington University in St. Louis Decisions are customarily a result of the Reviewing Editor and the peer reviewers coming together and discussing their recommendations until a consensus is reached. When revisions are invited, a fact-based synthesis statement explaining their decision and outlining what is needed to prepare a revision will be listed below. The following reviewer(s) agreed to reveal their identity: NONE.

The authors were highly responsive to the reviewer comments, and I believe the paper is ready to go as is! Two minor comments they may want to address:

1) The authors may want to include a graphical abstract

2) The authors may want to update the term “differential changes” in the title - I was unclear on what the changes were differential to as it was written.

## References

[B1] Adinoff B (2004) Neurobiologic processes in drug reward and addiction. Harv Rev Psychiatry 12:305–320. 10.1080/10673220490910844 15764467 PMC1920543

[B2] Akinola L, Mckiver B, Toma W, Zhu A, Tyndale R, Kumar V, Damaj M (2019) C57BL/6 substrain differences in pharmacological effects after acute and repeated nicotine administration. Brain Sci 9:244. 10.3390/brainsci9100244 PMC6827359 31546627

[B3] Benowitz NL, Hukkanen J, Jacob P 3rd (2009) Nicotine chemistry, metabolism, kinetics and biomarkers. Handb Exp Pharmacol (192):29–60. 10.1007/978-3-540-69248-5_2 PMC2953858 19184645

[B4] Bolton JL, Molet J, Regev L, Chen Y, Rismanchi N, Haddad E, Yang DZ, Obenaus A, Baram TZ (2018) Anhedonia following early-life adversity involves aberrant interaction of reward and anxiety circuits and is reversed by partial silencing of amygdala corticotropin-releasing hormone gene. Biol Psychiatry 83:137–147. 10.1016/j.biopsych.2017.08.023 29033027 PMC5723546

[B5] Brunzell D, Russell D, Picciotto M (2003) In vivo nicotine treatment regulates mesocorticolimbic CREB and ERK signaling in C57Bl/6J mice. J Neurochem 84:1431–1441. 10.1046/j.1471-4159.2003.01640.x 12614343

[B6] Chaffee B, Couch E, Gansky S (2017) Trends in characteristics and multi-product use among adolescents who use electronic cigarettes, United States 2011-2015. PLoS One 12:e0177073. 10.1371/journal.pone.0177073 28475634 PMC5419603

[B7] Ciocchi S, Herry C, Grenier F, Wolff SBE, Letzkus JJ, Vlachos I, Ehrlich I, Sprengel R, Deisseroth K, Stadler MB, Müller C, Lüthi A (2010) Encoding of conditioned fear in central amygdala inhibitory circuits. Nature 468:277–282. 10.1038/nature09559 21068837

[B8] Cooper S, Akers A, Henderson B (2021) Flavors enhance nicotine vapor self-administration in male mice. Nicotine Tob Res 23:566–572. 10.1093/ntr/ntaa165 32860507 PMC7885783

[B9] Cullen KA, Gentzke AS, Sawdey MD, Chang JT, Anic GM, Wang TW, Creamer MR, Jamal A, Ambrose BK, King BA (2019) e-Cigarette use among youth in the United States, 2019. JAMA 322:2095–2103. 10.1001/jama.2019.18387 31688912 PMC6865299

[B10] Dawkins L, Kimber C, Doig M, Feyerabend C, Corcoran O (2016) Self-titration by experienced e-cigarette users: blood nicotine delivery and subjective effects. Psychopharmacology (Berl) 233:2933–2941. 10.1007/s00213-016-4338-2 27235016

[B11] DeBaker M, Moen J, Robinson J, Wickman K, Lee A (2020) Unequal interactions between alcohol and nicotine co-consumption: suppression and enhancement of concurrent drug intake. Psychopharmacology (Berl) 237:967–978. 10.1007/s00213-019-05426-6 31858160 PMC7124972

[B12] De Biasi M, Dani J (2011) Reward, addiction, withdrawal to nicotine. Annu Rev Neurosci 34:105–130. 10.1146/annurev-neuro-061010-113734 21438686 PMC3137256

[B13] Douglass AM, Kucukdereli H, Ponserre M, Markovic M, Gründemann J, Strobel C, Alcala Morales PL, Conzelmann K-K, Lüthi A, Klein R (2017) Central amygdala circuits modulate food consumption through a positive-valence mechanism. Nat Neurosci 20:1384–1394. 10.1038/nn.4623 28825719

[B14] Franklin KB, Paxinos G (2008) The mouse brain in stereotaxic coordinates, compact. San Diego: Academic Press.

[B15] Freels TG, Baxter-Potter LN, Lugo JM, Glodosky NC, Wright HR, Baglot SL, Petrie GN, Yu Z, Clowers BH, Cuttler C, Fuchs RA, Hill MN, McLaughlin RJ (2020) Vaporized cannabis extracts have reinforcing properties and support conditioned drug-seeking behavior in rats. J Neurosci 40:1897–1908. 10.1523/JNEUROSCI.2416-19.2020 31953372 PMC7046447

[B16] Frie J, Underhill J, Zhao B, de Guglielmo G, Tyndale R, Khokhar J (2020) OpenVape: an open-source e-cigarette vapor exposure device for rodents. eNeuro 7:ENEURO.0279-20.2020. 10.1523/ENEURO.0279-20.2020 PMC7598908 32859723

[B17] Funk D, Coen K, Tamadon S, Hope B, Shaham Y, Lê A (2016) Role of central amygdala neuronal ensembles in incubation of nicotine craving. J Neurosci 36:8612–8623. 10.1523/JNEUROSCI.1505-16.2016 27535909 PMC4987435

[B18] Ghosh A, Coakley RC, Mascenik T, Rowell TR, Davis ES, Rogers K, Webster MJ, Dang H, Herring LE, Sassano MF, Livraghi-Butrico A, Van Buren SK, Graves LM, Herman MA, Randell SH, Alexis NE, Tarran R (2018) Chronic e-cigarette exposure alters the human bronchial epithelial proteome. Am J Respir Crit Care Med 198:67–76. 10.1164/rccm.201710-2033OC 29481290 PMC6034122

[B19] Ghosh A, Coakley RD, Ghio AJ, Muhlebach MS, Esther CR, Alexis NE, Tarran R (2019) Chronic e-cigarette use increases neutrophil elastase and matrix metalloprotease levels in the lung. Am J Respir Crit Care Med 200:1392–1401. 10.1164/rccm.201903-0615OC 31390877 PMC6884043

[B20] Gilpin N, Herman M, Roberto M (2015) The central amygdala as an integrative hub for anxiety and alcohol use disorders. Biol Psychiatry 77:859–869. 10.1016/j.biopsych.2014.09.008 25433901 PMC4398579

[B21] Glodosky NC, Cuttler C, Freels TG, Wright HR, Rojas MJ, Baglot SL, Hill MN, McLaughlin RJ (2020) Cannabis vapor self-administration elicits sex- and dose-specific alterations in stress reactivity in rats. Neurobiol Stress 13:100260. 10.1016/j.ynstr.2020.100260 33344714 PMC7739171

[B22] Gutierre ZA, Nguyen J, Creehan K, Taffe M (2020) Female rats self-administer heroin by vapor inhalation. Pharmacol Biochem Behav 199:173061. 10.1016/j.pbb.2020.173061 33164848 PMC7725943

[B23] Haubensak W, Kunwar PS, Cai H, Ciocchi S, Wall NR, Ponnusamy R, Biag J, Dong HW, Deisseroth K, Callaway EM, Fanselow MS, Lüthi A, Anderson DJ (2010) Genetic dissection of an amygdala microcircuit that gates conditioned fear. Nature 468:270–276. 10.1038/nature09553 21068836 PMC3597095

[B24] Herman M, Tarran R (2020) E-cigarettes, nicotine, the lung and the brain: multi-level cascading pathophysiology. J Physiol 598:5063–5071. 10.1113/JP278388 32515030 PMC7721976

[B25] Herman M, Contet C, Roberto M (2016) A functional switch in tonic GABA currents alters the output of central amygdala corticotropin releasing factor receptor-1 neurons following chronic ethanol exposure. J Neurosci 36:10729–10741. 10.1523/JNEUROSCI.1267-16.2016 27798128 PMC5083004

[B26] Hiler M, Breland A, Spindle T, Maloney S, Lipato T, Karaoghlanian N, Shihadeh A, Lopez A, Ramôa C, Eissenberg T (2017) Electronic cigarette user plasma nicotine concentration, puff topography, heart rate, and subjective effects: influence of liquid nicotine concentration and user experience. Exp Clin Psychopharmacol 25:380–392. 10.1037/pha0000140 29048187 PMC5657238

[B27] Javadi-Paydar M, Kerr TM, Harvey EL, Cole M, Taffe MA (2019) Effects of nicotine and THC vapor inhalation administered by an electronic nicotine delivery system (ENDS) in male rats. Drug Alcohol Depend 198:54–62. 10.1016/j.drugalcdep.2019.01.027 30878767 PMC6467722

[B28] Kasten C, Frazee A, Boehm S (2016) Developing a model of limited-access nicotine consumption in C57Bl/6J mice. Pharmacol Biochem Behav 148:28–37. 27242276 10.1016/j.pbb.2016.05.010 PMC4972646

[B29] Kenny P, Chartoff E, Roberto M, Carlezon W, Markou A (2009) NMDA receptors regulate nicotine-enhanced brain reward function and intravenous nicotine self-administration: role of the ventral tegmental area and central nucleus of the amygdala. Neuropsychopharmacology 34:266–281. 10.1038/npp.2008.58 18418357 PMC2654386

[B30] Koob G, Roberts A, Schulteis G, Parsons L, Heyser C, Hyytiä P, Merlo-Pich E, Weiss F (1998) Neurocircuitry targets in ethanol reward and dependence. Alcohol Clin Exp Res 22:3–9. 9514280

[B31] Kyerematen G, Owens G, Chattopadhyay B, deBethizy J, Vesell E (1988) Sexual dimorphism of nicotine metabolism and distribution in the rat. Studies in vivo and in vitro. Drug Metab Dispos 16:823–828. 2907460

[B32] Lallai V, Chen Y, Roybal M, Kotha E, Fowler J, Staben A, Cortez A, Fowler C (2021) Nicotine e-cigarette vapor inhalation and self-administration in a rodent model: sex- and nicotine delivery-specific effects on metabolism and behavior. Addict Biol. Advance online publication. Retrieved Feb 23, 2021. doi: 10.1111/adb.13024. PMC8380743 33624410

[B33] Lawson G, Hurt R, Dale L, Offord K, Croghan I, Schroeder D, Jiang N (1998) Application of urine nicotine and cotinine excretion rates to assessment of nicotine replacement in light, moderate, and heavy smokers undergoing transdermal therapy. J Clin Pharmacol 38:510–516. 9650540 10.1002/j.1552-4604.1998.tb05788.x

[B34] Lerner CA, Sundar IK, Yao H, Gerloff J, Ossip DJ, McIntosh S, Robinson R, Rahman I (2015) Vapors produced by electronic cigarettes and e-juices with flavorings induce toxicity, oxidative stress, and inflammatory response in lung epithelial cells and in mouse lung. PLoS One 10:e0116732. 10.1371/journal.pone.0116732 25658421 PMC4319729

[B35] LeSage M, Keyler D, Shoeman D, Raphael D, Collins G, Pentel P (2002) Continuous nicotine infusion reduces nicotine self-administration in rats with 23-h/day access to nicotine. Pharmacol Bioche Behav 72:279–289. 10.1016/s0091-3057(01)00775-4 11900798

[B36] Levin E, Rezvani A, Montoya D, Rose J, Swartzwelder H (2003) Adolescent-onset nicotine self-administration modeled in female rats. Psychopharmacology (Berl) 169:141–149. 10.1007/s00213-003-1486-y 12764575

[B37] Lüthi A, Lüscher C (2014) Pathological circuit function underlying addiction and anxiety disorders. Nat Neurosci 17:1635–1643. 10.1038/nn.3849 25402855

[B38] Markou A (2008) Review. Neurobiology of nicotine dependence. Philos Trans R Soc Lond B Biol Sci 363:3159–3168. 18640919 10.1098/rstb.2008.0095 PMC2607327

[B39] Montanari C, Kelley L, Kerr T, Cole M, Gilpin N (2020) Nicotine e-cigarette vapor inhalation effects on nicotine and cotinine plasma levels and somatic withdrawal signs in adult male Wistar rats. Psychopharmacology (Berl) 237:613–625. 10.1007/s00213-019-05400-2 31760460 PMC7039759

[B40] National Center for Chronic Disease Prevention and Health Promotion (US) Office on Smoking and Health (2016) E-cigarette use among youth and young adults: a report of the Surgeon General. Atlanta: Centers for Disease Control and Prevention. 30869850

[B41] O’Rourke K, Touchette J, Hartell E, Bade E, Lee A (2016) Voluntary co-consumption of alcohol and nicotine: effects of abstinence, intermittency, and withdrawal in mice. Neuropharmacology 109:236–246. 10.1016/j.neuropharm.2016.06.023 27342124 PMC5842373

[B42] Palazzolo D (2013) Electronic cigarettes and vaping: a new challenge in clinical medicine and public health. A literature review. Front Public Health 1:56. 10.3389/fpubh.2013.00056 24350225 PMC3859972

[B43] Picciotto MR, Kenny PJ (2013) Molecular mechanisms underlying behaviors related to nicotine addiction. Cold Spring Harb Perspect Med 3:a012112. 10.1101/cshperspect.a012112 23143843 PMC3530035

[B44] Picciotto MR, Mineur YS (2014) Molecules and circuits involved in nicotine addiction: the many faces of smoking. Neuropharmacology 76 [Pt B]:545–553. 10.1016/j.neuropharm.2013.04.028 23632083 PMC3772953

[B45] Pitkänen A, Amaral D (1994) The distribution of GABAergic cells, fibers, and terminals in the monkey amygdaloid complex: an immunohistochemical and in situ hybridization study. J Neurosci 14:2200–2224. 10.1523/JNEUROSCI.14-04-02200.1994 8158266 PMC6577158

[B46] Salminen O, Seppä T, Gäddnäs H, Ahtee L (1999) The effects of acute nicotine on the metabolism of dopamine and the expression of Fos protein in striatal and limbic brain areas of rats during chronic nicotine infusion and its withdrawal. J Neurosci 19:8145–8151. 10.1523/JNEUROSCI.19-18-08145.1999 10479714 PMC6782454

[B47] Smith LC, Kallupi M, Tieu L, Shankar K, Jaquish A, Barr J, Su Y, Velarde N, Sedighim S, Carrette LLG, Klodnicki M, Sun X, de Guglielmo G, George O (2020) Validation of a nicotine vapor self-administration model in rats with relevance to electronic cigarette use. Neuropsychopharmacology 45:1909–1919. 10.1038/s41386-020-0734-8 32544927 PMC7608444

[B48] Torres OV, Gentil LG, Natividad LA, Carcoba LM, O’Dell LE (2013) Behavioral, biochemical, and molecular indices of stress are enhanced in female versus male rats experiencing nicotine withdrawal. Front Psychiatry 4:38. 10.3389/fpsyt.2013.00038 23730292 PMC3657710

[B49] Valjent E, Pagès C, Hervé D, Girault J, Caboche J (2004) Addictive and non-addictive drugs induce distinct and specific patterns of ERK activation in mouse brain. Eur J Neurosci 19:1826–1836. 10.1111/j.1460-9568.2004.03278.x 15078556

[B50] Wang TW, Asman K, Gentzke AS, Cullen KA, Holder-Hayes E, Reyes-Guzman C, Jamal A, Neff L, King BA (2018) Tobacco product use among adults — United States, 2017. MMWR Morb Mortal Wkly Rep 67:1225–1232. 30408019 10.15585/mmwr.mm6744a2 PMC6223953

